# A randomized multicenter comparison of hybrid sirolimus-eluting stents with bioresorbable polymer versus everolimus-eluting stents with durable polymer in total coronary occlusion: rationale and design of the Primary Stenting of Occluded Native Coronary Arteries IV study

**DOI:** 10.1186/1745-6215-13-240

**Published:** 2012-12-15

**Authors:** Koen Teeuwen, Tom Adriaenssens, Ben JL Van den Branden, José PS Henriques, Rene J Van der Schaaf, Jacques J Koolen, Paul HMJ Vermeersch, Mike AR Bosschaert, Jan GP Tijssen, Maarten J Suttorp

**Affiliations:** 1 Department of Cardiology, St. Antonius Hospital, Koekoekslaan1, 3435 CM, Nieuwegein, the Netherlands; 2 Department of Cardiology, University Hospitals Leuven, Herestraat 49, 3000, Leuven, Belgium; 3 Department of Cardiology, Amphia Hospital, Molengracht 21, 4818 CK, Breda, the Netherlands; 4 Department of Cardiology, Academic Medical Center, University of Amsterdam, Meibergdreef 9, 1105 AZ, Amsterdam, the Netherlands; 5 Department of Cardiology, Onze Lieve Vrouwe Gasthuis, Oosterpark 9, 1091 AC, Amsterdam, the Netherlands; 6 Department of Cardiology, Catharina Hospital, Michalangelolaan 2, 6523 EJ, Eindhoven, the Netherlands; 7 Department of Cardiology, Middelheim Hospital, Lindendreef 1, 2020, Antwerpen, Belgium

**Keywords:** Chronic total occlusion, Drug-eluting stent, Angioplasty

## Abstract

**Background:**

Percutaneous recanalization of total coronary occlusion (TCO) was historically hampered by high rates of restenosis and reocclusions. The PRISON II trial demonstrated a significant restenosis reduction in patients treated with sirolimus-eluting stents compared with bare metal stents for TCO. Similar reductions in restenosis were observed with the second-generation zotarolimus-eluting stent and everolimus-eluting stent. Despite favorable anti-restenotic efficacy, safety concerns evolved after identifying an increased rate of very late stent thrombosis (VLST) with drug-eluting stents (DES) for the treatment of TCO. Late malapposition caused by hypersensitivity reactions and chronic inflammation was suggested as a probable cause of these VLST. New DES with bioresorbable polymer coatings were developed to address these safety concerns. No randomized trials have evaluated the efficacy and safety of the new-generation DES with bioresorbable polymers in patients treated for TCO.

**Methods/Design:**

The prospective, randomized, single-blinded, multicenter, non-inferiority PRISON IV trial was designed to evaluate the safety, efficacy, and angiographic outcome of hybrid sirolimus-eluting stents with bioresorbable polymers (Orsiro; Biotronik, Berlin, Germany) compared with everolimus-eluting stents with durable polymers (Xience Prime/Xpedition; Abbott Vascular, Santa Clara, CA, USA) in patients with successfully recanalized TCOs. In total, 330 patients have been randomly allocated to each treatment arm. Patients are eligible with estimated duration of TCO ≥4 weeks with evidence of ischemia in the supply area of the TCO. The primary endpoint is in-segment late luminal loss at 9-month follow-up angiography. Secondary angiographic endpoints include in-stent late luminal loss, minimal luminal diameter, percentage of diameter stenosis, in-stent and in-segment binary restenosis and reocclusions at 9-month follow-up. Additionally, optical coherence tomography is performed in the first 60 randomized patients at 9 months to assess neointima thickness, percentage of neointima coverage, and stent strut malapposition and coverage. Personnel blinded to the allocated treatment will review all angiographic and optical coherence assessments. Secondary clinical endpoints include major adverse cardiac events, clinically driven target vessel revascularization, target vessel failure and stent thrombosis to 5-year clinical follow-up. An independent clinical event committee blinded to the allocated treatment will review all clinical events.

**Trial registration:**

Clinical Trials.gov: NCT01516723. Patient recruitment started in February 2012.

## Background

Percutaneous coronary intervention (PCI) of total coronary occlusion (TCO) was limited by high rates of restenosis in patients treated with bare metal stents or plain old balloon angioplasty [1-4]. The introduction of drug-eluting stents (DES) demonstrated a significant reduction in restenosis and reocclusions [5,6]. The Primary Stenting of Occluded Native Coronary Arteries (PRISON) II trial was the first randomized trial in patients treated for TCOs that demonstrated a significant reduction in restenosis and reocclusions with sirolimus-eluting stents (SES) (Cypher; Cordis Corporation, Bridgewater, NY, USA) compared with bare metal stents [7]. The rate of in-segment binary restenosis was reduced from 41% to 11% in favor of SES. Clinical outcome remained significantly in favor of SES for up to 5 years, despite a higher rate of late and very late stent thrombosis (VLST) [8,9]. Second-generation DES were developed to address safety concerns for VLST observed in lesions treated with SES and paclitaxel-eluting stents [10-13].

In the PRISON III trial we compared two zotarolimus-eluting stents (ZES), the Endeavor ZES and the Resolute ZES (both Medtronic Inc., Minneapolis, MN, USA), with SES in patients treated for TCO [14]. Results after 1 year demonstrated similar efficacy and safety of the Resolute ZES compared with SES. However, the lesions treated with the Endeavor ZES showed a higher rate of late luminal loss, and a trend towards an increased rate of target lesion revascularization compared with SES. Furthermore, no stent thrombosis was observed in the Endeavor ZES or Resolute ZES groups, and only one possible stent thrombosis after SES implantation. Longer follow-up of this cohort is needed to observe the occurrence of VLST.

The everolimus-eluting stents (EES; Xience V/Xience Prime; Abbott Vascular, Santa Clara, CA, USA), also second-generation DES, showed a low rate of late luminal loss in the treatment of nonobstructive lesions with favorable clinical outcome [15]. The first results of EES in chronic total occlusions are encouraging and the CIBELES trial, which compares EES with SES in chronic total occlusions, is currently ongoing [16,17].

Despite the promising results of second-generation DES, new concerns have been raised after identifying hypersensitivity reactions caused by the durable polymer coatings of DES [18,19]. These reactions lead to chronic inflammation and possible late stent strut malapposition, which may lead to the development of VLST and delayed restenosis [20]. To address these limitations, new DES with bioresorbable polymer coatings have been developed. These polymers degrade gradually over the course of several months, resulting in controlled drug release without leaving inflammatory stimuli. A proof of concept for this new technology was recently demonstrated in the LEADERS trial [21]. The study was not powered for VLST. However, the group treated with biolimus-eluting stents with bioresorbable polymers showed a reduction of VLST by 80% relative risk at 4 years compared with conventional SES. Recently, a new hybrid sirolimus-eluting stent (Orsiro; Biotronik SE & Co. KG, Berlin, Germany) with bioresorbable polymers was developed. The first human study (BIOFLOW-I; Clinical Trial.gov: NCT01214148) in patients with *de novo* coronary lesions showed an excellent anti-restenotic effect on the 9-month angiography. Currently, the hybrid sirolimus-eluting stent is compared with EES in patients with *de novo* coronary lesions in the BIOFLOW-II study (Clinical Trial.gov: NCT01356888). Results are expected in 2013. Up to now there are no data available about the safety and efficacy of these newly developed DES with bioresorbable polymers in patients with TCO.

The PRISON IV trial is a prospective, randomized, single-blinded, multicenter trial, designed to evaluate the safety, efficacy, and angiographic outcome of hybrid sirolimus-eluting stents with bioresorbable polymers (Orsiro; Biotronik) compared with EES with durable polymers (Xience Prime/Xpedition; Abbott Vascular) in patients with successfully recanalized TCOs.

## Methods/Design

### Recruitment, enrolment and randomization

**Figure 1:**
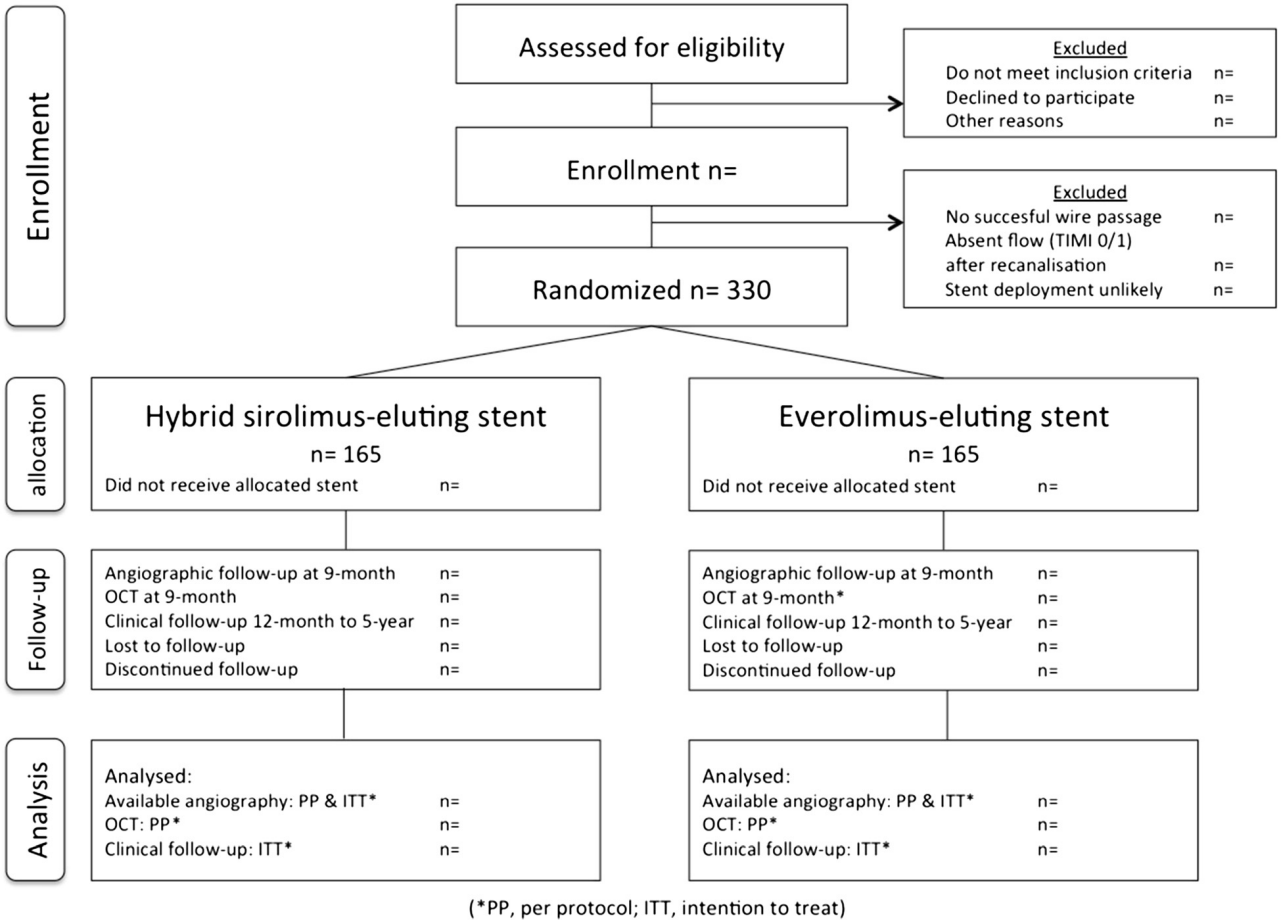
Study design.

In this study, a total of 330 patients are equally randomized for the treatment of TCOs with either a hybrid sirolimus-eluting stent (Orsiro; Biotronik) or an everolimus-eluting stent (Xience Prime/Xpedition; Abbott Vascular). The process of patient recruitment, enrolment and randomization is summarized in Figure 1. In detail, all patients presenting with stable coronary artery disease or acute coronary syndromes and the presence of TCOs on coronary angiography are assessed for study eligibility in eight high-volume PCI hospitals in the Netherlands and Belgium. Patients are eligible for study participation if the following criteria are met: the estimated duration of the TCO is ≥4 weeks; and there is evidence of myocardial ischemia in the supply area of the occluded artery. Evidence of myocardial ischemia should be demonstrated by either nuclear imaging, stress/perfusion magnetic resonance imaging or an exercise test (a positive exercise test is defined as electrocardiographic ST-depression ≥1.0 mm horizontal; downsloping or upsloping ST-depression ≥2.0 mm). Furthermore, the reference diameter of the target vessel should be between 2.25 and 4.0 mm, the lesion must successfully be crossed by a guide wire in the true distal lumen, and successful stent deployment should be assumable.

Patients are excluded if they are younger than 18 years, pregnant or nursing; if they are presented with total occlusions of previous venous or arterial bypass grafts; if they are known to have an allergy for the used anticoagulant or antiplatelet medication (clopidogrel, prasugrel, ticagrelor, acetylsalicylic acid and heparin) or the anti-proliferative agents (sirolimus and everolimus) mounted on the stents; or if life expectancy is less than 1 year or contributing factors make long-term follow-up not possible.

The randomization procedure is initiated after successful wire passage of the lesion. Randomization is performed using an interactive Web-based randomization system. After entering the patient’s sex, date of birth, randomization date and confirmation of eligibility, the randomization outcome is provided to the investigator/operator on the screen. Patients and referring physicians are blinded to the assigned treatment group. However, the operator is informed about the assigned stent.

#### Study oversight

The PRISON IV study is a prospective, randomized, multicenter clinical trial performed in six Dutch and two Belgian high-volume PCI hospitals. The Research and Development Department at the St. Antonius Hospital Nieuwegein is responsible for data collection and monitoring. The study authors are responsible for data analysis and interpretation. An independent clinical event adjudication committee will review all endpoints blinded. An independent Data and Safety Monitoring Board will review all cardiac and noncardiac adverse events unblinded, including acute kidney injury, contrast nephropathy and major bleeding. Serious adverse events (fatal, life-threatening, disabling or resulting in hospitalization or prolongation of hospitalization) are reported to the Data and Safety Monitoring Board within 24 hours.

The study is supported by an unrestricted grant from Biotronik SE & Co. KG. The sponsor has no role in the study design, data collection, analysis, interpretation of the data, drafting of the final manuscript, or the decision to submit the manuscript for publication. All institutional review boards of the local centers approved the study. Written informed consent is obtained prior to the procedure. The study is performed in compliance with the standards of Good Clinical Practice (ICH/E6/R1) and the Declaration of Helsinki (Washington 2002). The study is registered at Clinical Trials.gov number, NCT01516723.

#### Angiographic definitions

TCO is defined as an absence of antegrade flow of contrast distal to the occlusion (Thrombolysis and Myocardial Infarction flow 0 according to the Thrombolysis and Myocardial Infarction Grade flow) and only minimal flow of contrast distal to the occluded vessel (Thrombolysis and Myocardial Infarction Grade flow I) [22]. The duration of the coronary occlusion is at least 4 weeks and is estimated by clinical information. Chronic total occlusion is defined as a TCO with duration >3 months according to the guidelines on myocardial revascularization of the European Society of Cardiology [23]. The estimated length of the occlusion was measured from the proximal point of the total occlusion to the most distal point of the lesion, which was visualized with the first contrast injection after successful recanalization. The coronary segment is defined as the stented segment including the margins 5 mm distal and proximal to the stents.

#### Angioplasty procedure

The procedure is performed by either the radial or femoral approach with standard coronary catheterization techniques. The major goal is to achieve residual diameter stenosis <30% after stent implantation on visual assessment. Prior to the procedure, patients receive 5,000 to 10,000 international units heparin, followed by a bolus of 5,000 international units every additional hour of the procedure. Patients are equally assigned to the hybrid sirolimus-eluting stent or the everolimus-eluting stent. In the case of additional stents, only the assigned stent type is used per lesion and/or vessel. Post-stent dilatation is performed with high inflation pressures in all patients. All patients receive dual anti-platelet therapy for at least 12 months. Patients are preloaded with 300 mg aspirin and 300 to 600 mg clopidogrel at least 1 day before the procedure. Aspirin is continued at a once-daily dose of 80 to 100 mg lifelong and clopidogrel 75 mg once daily for at least 12 months. Operators are advised to follow the European Society of Cardiology guidelines on acute coronary syndromes concerning dual anti-platelet therapy, if patients are already treated with another PY12 inhibitor (ticagrelor 2× 90 mg or prasugrel 1×10 mg/1×5 mg; age >75 years or weight <60 kg) for a prior coronary event [24,25].

#### Quantitative coronary analysis

Coronary angiograms are digitally recorded at baseline, immediately after the procedure, and at 9-month follow-up, and are assessed offline by an independent angiographic core laboratory (St Antonius Hospital Angiographic Core Laboratory, Nieuwegein, the Netherlands) with an automatic edge-detection system (CMS version 5.3; Medis Medical Imaging Systems, Leiden, the Netherlands) by experienced personnel who are blinded for clinical information and type of stent implanted. Before angiography, 100 to 300 μg nitroglycerin is administered intracoronary. The nontapered tip of the catheter is used as the calibration standard. All lesions are assessed in at least two orthogonal views; the projection showing the smallest diameter (worst view) is used for quantitative coronary angiography analysis, and views with the least foreshortening are used for measuring the length of the occlusion. In disease-free proximal segments, the reference diameter is measured. Cineangiograms are obtained before, immediately after, and at 9 months using the same views at all times. Any coronary angiography performed within 6 months after the initial procedure is considered unscheduled. When an unscheduled angiography will be followed by target lesion or target vessel revascularization (TLR/TVR; either PCI or coronary artery bypass grafting), no further angiogram is needed. However, if no revascularization is performed at the repeated angiography, the scheduled 9-month angiography is still required. If the angiography takes place after 6 months, the 9-month angiography is omitted. Quantitative measurement will include the reference diameter of the vessel and the minimal lumen diameter. Of those measurements the following derived parameters are calculated: acute recoil, percentage of diameter stenosis, acute gain, late luminal loss, net luminal gain, and late loss index (late loss divided by the acute gain). Quantitative coronary analysis is used to evaluate the stented area (in-stent) and the area that included the stented segment as well as the 5-mm margins proximal and distal to the stent (in-segment). Angiographic binary in-stent restenosis is defined as ≥50% residual diameter stenosis within the stent. In-segment binary restenosis is defined as ≥50% residual diameter stenosis located in the stent and/or at the 5-mm proximal or 5-mm distal edge. Reocclusion is defined as a recurrent total occlusion at the previous angioplasty site.

#### Optical coherence tomography analysis

Optical coherence tomography (OCT) images are obtained in a total of 60 patients equally divided between the two stent groups during the follow-up angiogram at 9 months. The OCT procedures are performed in the first 60 patients treated in St. Antonius Hospital or UZ Leuven Hospital. All images are obtained with an optical frequency domain imaging system (C7XR OCT Imaging System; St Jude Medical, St Paul, MN, USA) and stored for offline analysis by an independent core laboratory at Leuven University Hospital (UZ Leuven Hospital, Medical Imaging Centre, Leuven, Belgium). Images are acquired with an automated pullback at a rate of 20 mm/second. Prior to the pullback, a bolus of 100 to 300 μg nitroglycerine is administered. The lens on the OCT wire is placed approximately 5 mm distal to the distal edge of the implanted stent and the pullback should include at least 5 mm of vessel proximal to the stent. All cross-sectional images (frames) are screened for quality assessment. Frames are excluded from analysis if any portion of the vessel is out of screen, if a side branch occupies >45° of the cross-section or if the image has poor quality caused by residual blood, sew-up artifacts, or reverberation. A dedicated automated software system developed at the Leuven Medical Imaging Centre (Odierna) is used for quantitative OCT analysis [26]. The following parameters are measured and/or calculated: percentage of malapposed stent struts; percentage of uncovered stent struts; tissue strut thickness (μm); neointimal hyperplasia; and absolute and percentage volume of intimal hyperplasia (mm^3^). Quantitative strut level analysis is performed every third frame (0.6 mm interval) along the entire target segment. The algorithm automatically determines the center of the luminal surface of the strut blooming and calculates its distance to the lumen contour. Stent strut malapposition is defined when this value is higher than the sum of strut thickness plus abluminal polymer thickness according to the stent manufacturer specifications plus a compensation of 20 μm to correct for strut blooming. The final cutoff value for malapposition is 90 μm for the hybrid sirolimus-eluting stent and 110 μm for the everolimus-eluting stent. A total percentage of malapposed struts is reported. If neointima is absent, a value of 0 is assigned. From these values, the total percentage of uncovered struts and the total volume of neointimal area are calculated. Furthermore, thickness of the neointimal coverage for each stent strut is reported.

#### Long-term follow-up

Clinical follow-up is performed at 1, 6 and 12 months and an annual evaluation up to 5 years. An independent clinical event committee, members of which are unaware of the patient’s treatment assignment, will review all clinical endpoints during follow-up. Recurrent angina, a positive exercise test, abnormal dobutamine stress magnetic resonance imaging and new perfusion defects on nuclear examination are considered clinical signs of restenosis. Follow-up angiography is performed earlier if there are clinical signs of restenosis, followed by revascularization when indicated. Percutaneous or surgical revascularization are regarded clinically driven if stenosis of the treated lesion was ≥50% of the lumen diameter on the basis of quantitative coronary angiography in the presence of ischemic signs or symptoms, or if there is diameter stenosis ≥70% irrespective of the presence or absence of ischemic signs or symptoms. Death, myocardial infarction (MI; defined as the presence of new significant Q waves or an elevation of creatine kinase or its MB isoenzyme to at least twice the upper limit) and clinically driven target lesion revascularization (TLR; defined as revascularization due to a stenosis within a 5 mm border proximal or distal to the stent) are recorded as major adverse cardiac events. Other secondary clinical endpoints include clinically driven TVR (defined as revascularization in the entire coronary vessel proximal and distal of the target lesion, including revascularization in side braches), target vessel failure (TVF; a composite of cardiac death, MI and clinically driven TVR) and stent thrombosis according to the definitions of the Academic Research Consortium. Cardiac death is defined as any death due to immediate cardiac cause (MI, low-output failure, fatal arrhythmia); death related to the procedure, nonwitnessed death, and death of unknown cause. Hypertension is defined as systolic blood pressure >140 mmHg and/or diastolic blood pressure >90 mmHg or use of antihypertensive drugs. Diabetes mellitus is defined as fasting venous glucose concentrations ≥7.8 mmol/l (140.5 mg/dl) or use of glucose-lowering drugs. Hypercholesterolemia is defined as fasting plasma cholesterol level >5.0 mmol/l (193 mg/dl) or use of cholesterol-lowering drugs. Finally, the occurrences of angina will be recorded in the Canadian Cardiovascular Society classification.

#### Endpoint definitions

The primary endpoint is in-segment late luminal loss at 9 months as assessed with quantitative coronary angiography. Secondary angiographic endpoints include the following: in-stent late luminal loss, acute recoil, acute gain, net luminal gain, late loss index, minimal lumen diameter, percentage of diameter stenosis, in-stent and in-segment binary restenosis and reocclusions at 9-month angiographic follow-up. Secondary clinical endpoints include a composite of major adverse cardiac events (death, MI and clinically driven target lesion revascularization), clinically driven TVR, TVF (cardiac death, MI, clinically driven TVR) and stent thrombosis up to 5 years of clinical follow-up. Tertiary OCT endpoints at 9-month follow-up are the following: percentage of uncovered stent struts, percentage of malapposed stent struts, tissue strut thickness, and absolute and percentage intimal hyperplasia. According to the new Academic Research Consortium definitions, stent thrombosis is defined as definitive if there is angiographic documentation of either occlusion of the target lesion or thrombus within or adjacent to a previously stented segment. Stent thrombosis is defined as probable if sudden unexplained death occurs within 30 days or if target vessel MI occurs without angiographic documentation, and is defined as possible if a sudden unexplained death that could not be attributed to another cause occurs after 30 days.

#### Statistical analysis

The objective of the study is to assess whether the outcome of treatment with the hybrid sirolimus-eluting stent is non-inferior to the outcome of treatment with the everolimus-eluting stent in patients with TCOs. The non-inferiority margin is set at the conventional level of 0.2 mm. The null hypothesis of inferiority of the hybrid sirolimus-eluting stent (Orsiro; Biotronik) is rejected when the upper limit of the 95% confidence interval for the observed difference in late loss falls below 0.2 mm. For the sample size calculation, we assumed that the mean late luminal loss is equal at 0.16 mm [15] in both treatment groups, with a common standard deviation of 0.55 mm. With 131 patients per treatment arm, the study has 90% power to reject the null hypothesis of inferiority of the hybrid sirolimus-eluting stent (Orsiro; Biotronik) relative to the everolimus-eluting stent (Xience Prime/Xpedition; Abbott Vascular). Analyzable follow-up angiograms are expected to be available for approximately 80% of the patients. Therefore, 165 patients per treatment arm are included in this study. Sample size calculations were performed with the use of the NQUERY computer program (Statistical Solutions, Saugus, MA, USA). The primary endpoint and other angiographic endpoints are both analyzed per protocol and on an intention-to-treat principle. No formal statistics are performed on clinical endpoints. Analysis of clinical endpoints is performed for descriptive purposes only on an intention-to-treat principle. The analysis of the OCT data is performed in an exploratory context.

## Conclusions

The PRISON II and PRISON III studies have shown favorable angiographic results for both the sirolimus-eluting stent and the Resolute zotarolimus-eluting stent for the treatment of TCOs. Recently, the everolimus-eluting stent with durable polymers also showed promising results in patients with chronic total occlusions. However, new DES with a bioresorbable polymer coating have emerged. At the moment, none of these stents with bioresorbable polymers have been evaluated in complex lesions such as TCO. The PRISON IV study evaluates the efficacy, safety, and angiographic outcome of the hybrid sirolimus-eluting stents with bioresorbable polymers compared with EES with durable polymers in successfully recanalized TCOs.

### Trial status

Recruitment started in February 2012.

## Abbreviations

DES: Drug-eluting stents; EES: Everolimus-eluting stents; MI: Myocardial infarction; OCT: Optical coherence tomography; PCI: Percutaneous coronary intervention; PRISON: Primary Stenting of Occluded Native Coronary Arteries; SES: Sirolimus-eluting stents; TCO: Total coronary occlusion; TVR: Target vessel revascularization; VLST: Very late stent thrombosis; ZES: Zotarolimus-eluting stents.

## Competing interests

The authors declare that they have no competing interests.

## Authors’ contributions

MJS, RJVdS, JJK and PHMJV were involved in the conception and design of the study. KT, TA, BJLVdB and MARB contributed to the drafting and writing of the manuscript. JGPT performed statistical considerations and power calculations. JPSH, JGPT and MJS critically revised the manuscript for intellectual content, with final approval for submission of the principal investigator MJS. All authors read and approved the manuscript.
